# Prognostic Value of Liver and Spleen Stiffness in Patients with Fontan Associated Liver Disease (FALD): A Case Series with Histopathologic Comparison

**DOI:** 10.3390/jcdd8030030

**Published:** 2021-03-16

**Authors:** Massimo A. Padalino, Liliana Chemello, Luisa Cavalletto, Annalisa Angelini, Marny Fedrigo

**Affiliations:** 1 Pediatric and Congenital Cardiac Surgery Unit, Department of Cardiac, Thoracic and Vascular Sciences and Public Health, University of Padova Medical School, 35128 Padova, Italy; 2 Internal Medicine and Hepatology Unit, Clinica Medica 5, Department of Medicine-DIMED, University of Padova Medical School, 35128 Padova, Italy; luisa.cavalletto@unipd.it; 3 Cardiovascular Pathology Unit, Department of Cardiac, Thoracic and Vascular Sciences and Public Health, University of Padova Medical School, 35128 Padova, Italy; annalisa.angelini@unipd.it (A.A.); marny.fedrigo@aopd.veneto.it (M.F.)

**Keywords:** FALD, Fontan failure, cirrhosis, transient elastography, liver stiffness, liver histology

## Abstract

The Fontan operation is the current surgical procedure to treat single-ventricle congenital heart disease, by splitting the systemic and pulmonary circulations and thus permitting lifespan to adulthood for the majority of newborns. However, emerging data are showing that Fontan-associated liver disease (FALD) is an increasing related cause of morbidity and mortality in patients with the Fontan circuit. We described the clinical, laboratory, and transient elastography (TE) findings in a case series of adults with the Fontan circuit, and also correlated data with post-mortem histological features, aimed to define the prognostic value of TE in the staging of FALD. All patients presented signs of a long-standing Fontan failure, characterized by reoperation need, systemic ventricle dysfunction, and FALD stigmata (liver and spleen enlargement, portal vein and inferior vena cava dilation, and abnormal liver function tests). Liver and spleen stiffness (LS and SS) values were indicative of significant liver fibrosis/cirrhosis and the presence of suggestive portal hypertension (LS mean 35.9; range 27.3–44.7 kPa; SS mean 42.1, range 32.2–54.5 kPa). Post-mortem evaluations confirmed a gross hepatic architecture distortion in all cases. All patients died from severe complications related to liver dysfunction and bleeding. TE correlated well with pathological findings and FALD severity. We propose this validated and harmless technique to monitor liver fibrosis extension and portal hypertension over time in Fontan patients, and to identify the optimal timing for surgical reoperations or orthotopic-heart transplantation (OHT), avoiding a higher risk of morbidity and mortality in cases with severe FALD.

## 1. Introduction

Congenital heart diseases (CHDs) are rare conditions, with a reported incidence of 2–8/1000 live births, which need surgical repair in early infancy. The Fontan operation is a widely used surgical procedure that consistently extends the lifespan of cases with single-ventricle (SV) anatomy [1]. The Fontan circuit delivers systemic venous blood directly to pulmonary arteries in cases with SV, separating the pulmonary and systemic circulations, suddenly improving oxygen saturation in the blood. However, this pseudo-circulation increases the central venous pressure and decreases cardiac output, leading to several long-term complications such as protein-losing enteropathy (PLE), plastic bronchitis, arrhythmias, heart failure, and hepatic fibrosis [2].

In particular, long-term liver congestion causes the development of cirrhosis and related complications (ascites, spontaneous bacterial peritonitis, encephalopathy, hepato-renal syndrome, gastro-esophageal bleeding, and hepatocellular carcinoma (HCC)) that are increasing incidence of morbidity and mortality related to Fontan correction [3]. Even if liver injury appears to be multifactorial, because of different noxae (hypoxemia, ischemia-reperfusion, thrombosis, infections, or drug-toxicity) during multiple surgeries, the Fontan circuit creates the condition of venous hepatic stasis, with central vein and sinusoid dilation, abnormal liver perfusion, and a slow-down of portal blood flow. These unexpected and often misrecognized changes, related to the Fontan circuit, have been recently defined as Fontan-associated liver disease (FALD) [4].

We report here the clinical events in four adult patients with SV and Fontan correction who underwent redo-surgery for a failing Fontan, and who died from liver dysfunction and bleeding. We compared the non-invasive transient elastography (TE) with the post-mortem pathological features in order to define its prognostic value.

## 2. Materials and Methods

We considered four consecutive patients (three males, aged 22, 23, and 27 years and one female 50 years old) with SV and Fontan-circuit who presented with a long clinical period characterized by reoperation need, systemic ventricle dysfunction, and the presence of FALD signs (i.e., liver and spleen enlargement, portal and inferior vena cava (IVC) dilation, and abnormal liver function tests), who died soon after a surgical procedure or heart transplantation from complications related to an underestimated chronic liver disease.

All patients underwent an abdominal US with a Doppler analysis to investigate the echostructure and echogenicity of the liver, spleen, and kidney parenchyma and to detect any signs of portal hypertension (i.e., measuring spleen and portal vein Ø and flow velocity rate) or systemic venous hypertension (i.e., measuring Ø of IVC and suprahepatic veins). The main echocardiographic parameters were also considered and calculated for the classification of the functional heart performances and NYHA stage (i.e., measuring telediastolic ventricular volume, stroke volume, cardiac output, ejection fraction, IVC Ø, and collapsibility). The liver and spleen stiffness (LS and SS) were obtained using a FibroScan^®^ (Echosens, Paris, France) following a standardized procedure, using an M probe, reporting values in kiloPascal (kPa; range 1.5–75 and 6.0–100, respectively) and with an interquartile range under 30%.

A complete autopsy was performed according to the standard protocol adopted in our centre and previously published both for post-transplant [5] or adult CHD [6] cases. Histopathology was part of the complete autopsy, and the liver samples were collected and stained with Haematoxylin–Eosin and Masson’s Trichromic (Diapath S.p.A., Martinengo (BG), Italy) for evaluation both of the extracellular matrix and fibrous pattern. Histopathologic semiquantitative methods were applied according to the morphological study by Kendall TJ et al. [7], particularly for the description of fibrosis deposition, sinusoids dilation, and hepatocytes/bile ducts damage, using the Metavir score [8] for inflammatory activity grading and fibrosis staging, and specially the congestive hepatic fibrosis (CHF) score [9] for grading the congestive (related to heart acute and chronic failure) and fibrotic (reactive to chronic liver damage) components.

## 3. Results

The clinical characteristics and surgical corrections of the patients are detailed in Table 1. Three cases showed a left systemic ventricle, and had major defects such as tricuspid atresia (TA) or double-inlet left ventricle (DILV) with transposition of great arteries (TGA) and pulmonary artery (PA) stenosis or aorta coarctation, and the other patient had hypoplastic left heart syndrome (HLHS). Only one case received a lateral-tunnel at 3 years of age, while the others were corrected with a cavopulmonary at 20 and 15 years, or an atriopulmonary connection at 16 years of age—this case also received a redo-Fontan at 41-years. All cases needed reoperation: one for a pseudo-aneurism resection, one for a LVAD implantation, one for an ascending aorta replacement, and the last case received an orthotopic-heart transplantation (OHT).

All cases have suffered from severe depression of the SV pump (ejection fraction (EF) < 47%; three cases NYHA class III and one IV), and showed failing Fontan signs (two with chronic cyanosis and impaired kidney function needing dialysis, and the remaining one with PLE and arrhythmia). All cases presented episodes of abdominal ascites and oedema. In the lab tests, all had only a slight abnormal increase of bilirubin and GGT, but only one of AST, and no one of ALT. Three out of four cases showed initial thrombocytopenia and all lymphopenia, and scored a CPT class B8 or B9. Upon medical examination, patients documented an enlargement of liver and spleen, and upon abdominal-US they showed dilation of IVC and, in three cases, of the portal vein as well. At post-mortem pathological examination, all cases showed a gross architectural distortion of the hepatic lobule (grade 3 or 4) with sinusoidal dilation (grade 2 or 3) and a CHF score [9] ranging from 2B to 4, respectively, with the Metavir [8] score being F3 in Case 1 but F4 in the remaining ones.

**Figure 1:**
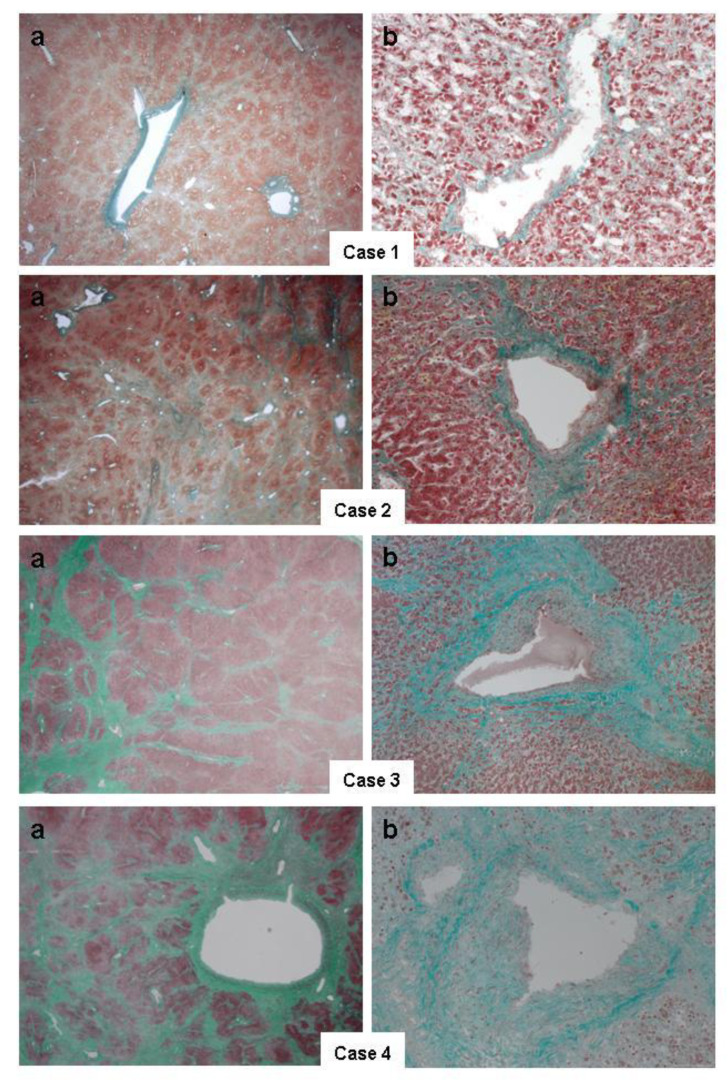
Liver histopathology obtained at autoptic examination by Masson’s Trichromic staining: (**a**) a panoramic view (×10 magnification) and (**b**) close up view (×40 magnification). In particular, the marked distortion of the liver structure, with fibrotic bridges arising from centrilobular veins and involving sinusoidal spaces and portal tracts, can be seen. Note the increasing thickness of fibrotic septa from Cases 1 to 4, so as to manifest the characteristic condition of “reverse cirrhosis”; moreover, in Case 4, the presence of cholestasis also resembles the so called “nutmeg pattern”.

In Figure 1, all cases showed a marked distortion of liver architecture, with fibrotic bridges arising from centrilobular vein and involving sinusoidal spaces and portal tracts, so as to manifest the tipical condition of “reverse cirrhosis”. In particular, Case 1, staged Metavir F3 with a CHF score 2B, showed a mild fibrosis of the centrilobular veins and periportal tract, and delicate less extensive fibrosis of the sinusoidal spaces. In Case 2, the centrilobular fibrosis appeared to be thicker, with evident fibrotic bridging septa among the central veins and with a consistent sinusoidal fibrosis deposition. In Cases 3 and 4, was documented a gross architectural distortion of the liver, with diffuse sinusoidal fibrosis, and large septa (both with a Metavir of F4 and CHF scores of 3 and 4, respectively; see Table 2). In Case 4, there was also severe cholestasis, resembling the so called “nutmeg pattern”.

Table 2 details the pathological features and the cause of death for the reported cases. Current and severe liver disease mainly contributed to the unfavourable fate and death in all patients. Very abnormal values of LS and SS (mean values of 35.9 and 46.9 kPa, respectively) were obtained by TE. Furthermore, LS correctly identified and graded the FALD, which was in accordance with the post-mortem pathological features, in order to fully satisfy its diagnostic and accurate role for all cases. In addition, SS was related to the presence of clinical significant portal hypertension and even bleeding risk at values over 50 kPa, as evidenced in Cases 2 and 3.

## 4. Discussion

In this case series with SV-CHD and prolonged failing Fontan, the common finding of FALD represented the main comorbidity and the cause of death for all patients.

The prevalence of FALD is not well defined yet. Indeed, liver deterioration probably starts immediately after Fontan surgery [10], with the contribution of iatrogenic damage. However, this status often remains hidden for years, with the only evidence of liver venous congestion and stasis, revealing itself after many complications due to liver cirrhosis.

Post-Fontan liver histology has been reviewed in multiple small case series, including autopsy reviews and needle liver biopsies [3,11]. The histological analyses generally show lobular hepatic congestion and fibrotic distortion of the liver architecture in the majority of them. Although the liver biopsy is certainly a highly valuable diagnostic procedure, it is hardly applicable in the Fontan cohort, because of the high venous pressure and very common use of anticoagulation therapy—both conditions with a high risk of bleeding. In our cases, the histological features were performed according to various semiquantitative analyses [7,8,9]. In particular, Metavir [8] described a severe stage of liver cirrhosis in three out of four cases, and the CHF score [9] also confirmed a congestive pattern of grade 3 and 4 in these subjects (Cases 2–4).

On the other hand, we can rely in multiple non-invasive diagnostic imaging methods that can identify changes in the liver parenchyma (i.e., increased echogenicity, uneven texture, or liver surface nodularity), or can discover complications related to liver cirrhosis (i.e., liver and spleen enlargement, collateral circles, ascites, and hypervascular or HCC nodules) with sufficient specificity and accuracy [12]. The frequency of abnormal US findings has been shown to increase with time after the Fontan procedure [10], but contradictory correlations have been found between abnormal liver structures and biochemical parameters, hemodynamic data, age at Fontan, and other underlying diagnoses or ventricular morphologies.

Here, the long duration of Fontan failure and its determining role on the increase of central venous pressure are certainly conditions that can be related to the entity of FALD in at least two cases (Cases 3 and 4). On the other hand, in the remaining two patients, where Fontan circulation was more recent (Cases 1 and 2), prolonged hypoxia may have played a main role in the progression of FALD. In fact, the hypoxic liver injury, also known as hypoxic hepatitis, has been described long time ago [13], caused by an inadequate oxygen uptake by the centrilobular hepatocytes, resulting in necrosis and residual fibrosis. This hypoxic insult can also affect nephrons and promote the development of ischemic acute tubular necrosis and kidney failure with the need for dialysis (as for Cases 1 and 2). Only one patient (Case 3) reported in the medical history a previous acute hepatitis C with spontaneous viral clearance within 6 months from the acute phase of infection. Thus, the major pathogenetic mechanism of FALD in these cases can be considered to be heart pump failure, at preload and after load site, which induces both sinusoidal stasis and ischemia in major organs during the failing Fontan [2,4].

Recently, TE has been shown to be a promising novel non-invasive tool for long-term follow-up of cases with Fontan circuit [1,14]. In our case series, all patients had clinical evidences and TE data that were confirmatory of FALD. In addition, the evaluation by TE proved to be in agreement with the pathologic findings and this certainly strengthened its accurate role in staging liver fibrosis and portal hypertension. The LS showed elevated in all cases (mean of 35.9 kPa; range: 27.3–44.7 kPa), although common liver function tests (i.e., AST, GGT, and bilirubin) did not appear severely abnormal, and even the MELD-XI scores ranged low values as 11–13. However, LS is clearly related even to liver congestion, proven by IVC dilation in all cases, as well as to gross fibrotic lobular distortion, which develops over time. This is confirmed in the current literature, where a progressive LS increase in relation to a longer time from Fontan is described [8,10].

**Figure 2:**
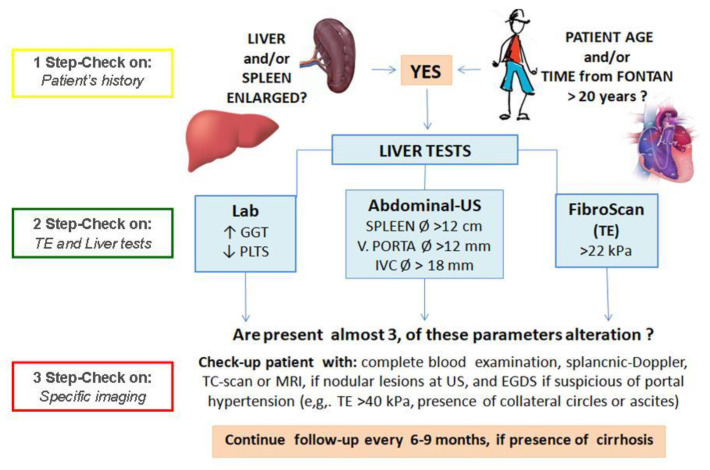
Step-by-step laboratory and instrumental approach to manage cases with signs or characteristics suggestive of Fontan-associated liver disease (FALD) in cases with single-ventricle (SV) and Fontan circuit.

Therefore, we believe, that TE still remains a practical and easy method to non-invasively distinguish between cases with and without progressive FALD. We found the LS follow-up to be a very useful and prognostic tool to monitor the related signs to Fontan circulation failing [15] and liver damage promotion in cases with Fontan circuit [16], particularly when found over the cut-off value of 22 kPa. Moreover, higher SS values appeared significantly related to the bleeding risk (as in Cases 2 and 3) [17]. Thus, we propose introducing TE for the evaluation of both congestion and fibrosis components of the liver and for the definition of portal hypertension, as part of the routine Fontan patients’ clinical follow-up (see Figure 2), reserving a secondary role of the liver biopsy in this peculiar cohort.

## 5. Conclusions

FALD is a serious and life threatening condition during long-term outcomes after the Fontan operation, and its urgently needs an accurate evaluation as a combined heart–liver transplantation may be required in selected cases. In our experience, TE has been proven to be a simple, accurate, and non-invasive method to monitor the stage of FALD through measurement of LS, but also to indicate Fontan circuit dysfunction early on. In addition, SS has revealed its diagnostic accuracy for the evaluation of portal hypertension and prediction of bleeding risk. Thus, we believe that all patients with major congenital heart disease and Fontan circuit should routinely undergo TE evaluation as part of their regular cardiologic follow-up in order to reveal hidden clinical conditions, especially liver related, and to possibly prevent postoperative complications that reduce patient survival.

## Figures and Tables

**Table 1:** Clinical and laboratory characteristics at the pre-operative presentation of cases.

Clinical Characteristics	Case-1 ♂	Case-2 ♂	Case-3 ♀	Case-4 ♂
Type of CHD	HLHS	TA + TGA + CoA	DILV + TGA + PA stenosis	TA + TGA Sub-PA stenosis
Type of sistemic ventricle	righ	left	left	left
Age at first Fontan, years	20	25	16	3
Type of Fontan	TCPC	TCPC	Atrio pulmonary	Lateral-tunnel
Time from Fontan, years	2	2	34	20
Redo Fontan	no	no	TCPC + BDG + Ablation	no
Age at last reoperation	22	27	50	23
Type of reoperation	Pseudo- aneurysm resection	Fenestration closure + LVAD implantation	OHT	Sub-Ao stenosis resection + AVR + Asc.Aorta replacement
Pre-operative NYHA class	III	III	III	IV
Ejection fraction (EF) (<55%)	**47%**	**37%**	**38%**	**36%**
Protein-losing enteropathy	no	no	**yes**	no
Dialysis session (>3 mos)	**yes**	**yes**	no	no
Pre-operative arrhythmias	no	no	**yes**	no
Pre-operative cyanosis	**yes**	**yes**	no	no
**Pre-Operative Laboratory Parameters (Normal Values)**				
PLTS, ×10^3^ (150–450)	200	**148**	**148**	**142**
Lymphopenia, mm3 (<1.1)	**yes**	**yes**	**yes**	**yes**
PT (INR) (0.9–1.2)	1.18	**1.91**	**1.66**	**1.36**
AST/ALT/GGT, IU/L (<45)	**48**/27/**82**	36/28/**99**	26/38/**66**	35/26/**56**
Bilirubin, umol/L (1.7–17)	**30**	**23**	**28**	**38**
Albumin, g/L (4.2–5)	**28**	**32**	**28**	**30**
Creatinine, umol/L (45–82)	130	115	105	90
eGFR, ml/min/sqm (>90)	**48**	**50**	**62**	80
CPT/MELD-XI score	B8/13	B9/12	B9/11	B8/11
**Pre-Operative Clinical and Instrumental Data**				
Liver, enlarged and stiff	**yes**	**yes**	**yes**	**yes**
Spleen Ø, cm (normal < 12)	**13**	**19**	**17**	**19**
Portal vein Ø, mm (<12)	**12**	**15**	**14**	**15**
IVC Ø, mm (<17)	**24**	**25**	**26**	**28**
Ascites decompensation	**yes**	**yes**	**yes**	**yes**

HLHS—hypoplastic left heart syndrome; TA—tricuspid atresia; TGA—transposition of great arteries; CoA—aortic coarctation; DILV—double-inlet left ventricle; Ao—aorta; PA—pulmonary artery; AVR—aortic valve replacement; BDG—bi-directional Glen; LVAD––left ventricular assist device; OHT—orthotopic-heart transplantation; TCPC—total cavopulmonary connection. Clinical and lab parameters in bold characters are abnormal.

**Table 2:** Description of the type and cause of death in relation to liver post-mortem pathological features and to transient elastography (TE) measurements.

Patient	Case-1 ♂	Case-2 ♂	Case-3 ♀	Case-4 ♂
Age at death, years	22	27	50	23
Time of death from reoperation, days	39	37	38	33
**Type and Cause of Death**				
Clinical type of death	Sudden respiratory arrest, after sedation for CVA positioning	Acute on chronic liver failure and hemorragic shock	Acute on chronic liver failure and hemorragic shock	Acute on chronic liver failure and intravasal coagulation
Pathological cause of death	FALD with multiorgan deterioration	Severe FALD with multifocal bleeding and multiorgan damage	Severe FALD with enteric hemorrhage and multiorgan damage	Severe FALD with multiorgan deterioration
**Histopathological Features** [7] at Post Mortem Examination of Liver *				
Gross architecture distortion (0–4)	3	4	4	4
Sinusoidal fibrosis (0–3)	1	2	2	3
Sinusoidal dilation (0–3)	2	2	2	3
Bile duct reaction (0–3)	1	1	2	1
Cholestasis (0–2)	1	1	1	2
Metavir score [8]	F3	F4	F4	F4
Congestive hepatic fibrosis (CHF) score [9]	2B	3	3	4
**Transient Elastography by Fibroscan**				
Liver stiffness, kPa (range 1.5–75 kPa)	27.3	37.4	34.4	44.7
Spleen stiffness, kPa (range 6.0–100 kPa)	30.6	56.4	52.2	48.7

CVA—central venous access; * Hepatocellular damage in autoptic cases was not evaluable. All cases showed peri-sinusoidal and peri-venular fibrosis, but none showed iron deposition or inflammation.

## Data Availability

Not applicable.
